# D-Dimer Can Serve as a Prognostic and Predictive Biomarker for Metastatic Gastric Cancer Treated by Chemotherapy

**DOI:** 10.1097/MD.0000000000000951

**Published:** 2015-07-31

**Authors:** Se-Il Go, Min Jeong Lee, Won Sup Lee, Hye Jung Choi, Un Seok Lee, Rock Bum Kim, Myoung Hee Kang, Hoon-Gu Kim, Gyeong-Won Lee, Jung Hun Kang, Jeong-Hee Lee, Sun Joo Kim

**Affiliations:** From the Department of Internal Medicine, Institute of Health Sciences, Gyeongsang National University School of Medicine, Jinju, Korea (S-IG, MJL, WSL, HJC, USL, MHK, H-GK, G-WL, JHK); Department of Preventive Medicine and Environmental Health Center, Gyeongsang National University School of Medicine, Jinju, Korea (RBK), Department of Pathology, Institute of Health Sciences, Gyeongsang National University School of Medicine, Jinju, Korea (JHL); and Department of Laboratory Medicine, Institute of Health Sciences, Gyeongsang National University School of Medicine, Jinju, Korea (SJK).

## Abstract

Systemic activation of hemostasis and thrombosis has been implicated in tumor progression and metastasis. D-dimer has been used as an indicator for the thrombosis. Here, we investigated the role of the activation of coagulation in patients with metastatic gastric cancer by measuring D-dimer level.

We conducted an observation study of 46 metastatic gastric cancer patients who received palliative chemotherapy (CTx). D-dimer levels were assessed before CTx and at the first response evaluation after CTx.

The overall survival (OS) of patients with pretreatment D-dimer levels <1.5 μg/mL was significantly longer than that of patients with D-dimer levels ≥1.5 μg/mL (22.0 vs 7.9 months, *P* = 0.019). At the first response evaluation, the mean level of D-dimer was significantly decreased by 2.11 μg/mL in patients either with partial response or stable disease (*P* = 0.011) whereas the mean level of D-dimer, although the difference did not reach statistical significance, was increased by 2.46 μg/mL in patients with progressive disease. In addition, the OS of patients with D-dimer levels <1.0 μg/mL at the first response evaluation was significantly longer than that of patients with D-dimer levels ≥1.0 μg/mL (22.0 vs 7.0 months, *P* = 0.009). The lower D-dimer levels (<1.0 μg/mL) at the first response evaluation after CTx was independent predictive factor for better survival in multivariate analysis (*P* *=* 0.037).

This study suggests that D-dimer levels may serve as a biomarker for response to CTx and OS in patients with metastatic gastric cancer.

## INTRODUCTION

Gastric cancer is the most common cancer and the third leading cause of cancer death in Korea.^1^ For localized gastric cancer, surgical resection is the curative therapy at present. However, a significant number of patients with gastric cancer experience a recurrence after surgery or are firstly diagnosed with metastatic disease. In this case, systemic chemotherapy (CTx) is a standard treatment, but the prognosis is very poor.^2^ Recently, comprehensive molecular characterization of gastric cancer enables to conduct tailored therapy that will improve survival of the patients with gastric cancer.^3^ However, there are few prognostic or predictive biomarkers for the treatment of gastric cancer. For the operable cases, tumor node metastasis (TNM) staging is the most important for predicting prognosis of gastric cancer so far, but for the metastatic gastric cancer, TNM staging cannot serve as a prognostic factor anymore.^4^ Now, carcinoembryonic antigen (CEA) is widely used as a monitoring tool for cancer progression of gastric cancer after surgery.^5^ However, only few data that needs more validation are available.

**FIGURE 1:**
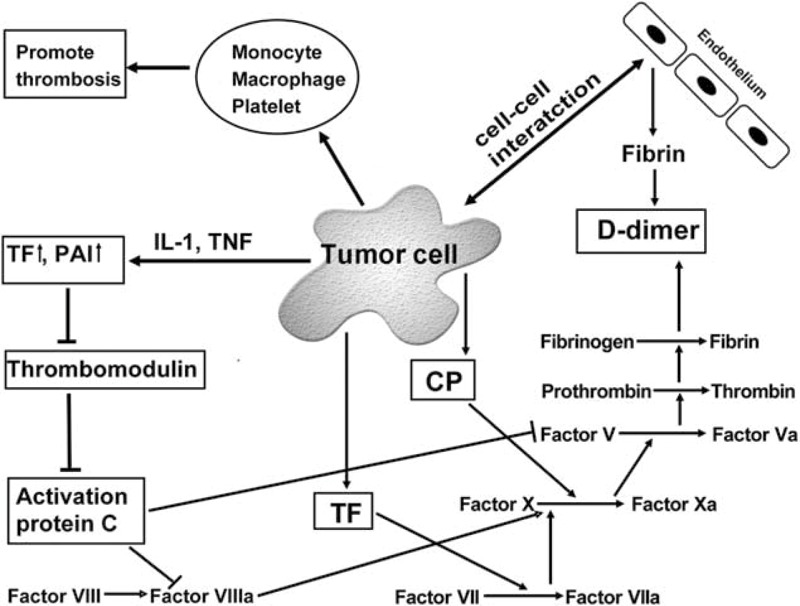
Mechanisms for cancer-induced hypercoagulation and consequential D-dimer formation. Cancer cells promote a hypercoagulable status and activate the hemostatic system. The cancer cells induce the hypercoagulable status by cell-to-cell interaction with endothelial cells, direct release of TF and CP, production of cytokines such as IL-1and TNF, and activation of monocyte, macrophage, and platelet. CP = cancer procoagulants, IL-1 = interleukin-1, PAI = plasminogen activator inhibitor, TF = tissue factor, TNF = tumor necrosis factor.

The cause of cancer death is mostly due to cancer progression, but thromboembolism also accounts for 10% to 20%.^6^ Levitan et al^7^ reported that the risk for venous thromboembolism (VTE) in cancer patients is 6 times higher than the control group. It is well known that gastric cancer has a high risk of developing VTE in particular.^8^ VTE in cancer patients is typically associated with plasma hypercoagulability, endothelial damage, and stasis of blood flow.^9^ The possible mechanisms of hypercoagulability are shown in Figure 1. The clotting process is exacerbated by direct interaction between cancer cells and the endothelial cells, by activating blood cells such as monocyte, macrophage, and platelet, and/or by secreting tissue factor, cancer procoagulants, and cytokines from cancer cells.^10,11^ Interestingly, these coagulation products are also associated with the growth, progression, metastasis, and angiogenesis of cancer.^11,12^ Therefore, thromboembolism is not only a direct cause of death in cancer patients but also closely related to the death from cancer progression. This is supported by the reports that the coagulation abnormality is associated with low survival rate in cancer patients.^8,13^

As a compensation mechanism for fibrin clot formation, fibrinolysis is activated, and then D-dimer is produced. The D-dimer is a specific indicator that reflects degradation of cross-linked fibrin polymer by plasmin and is widely used as assessment tool for diagnosis and treatment of thrombosis.^14^ The role of D-dimer as a prognostic factor has been evaluated mostly in operable colorectal, pancreatic, and lung cancer.^15–17^ Given the suggestion that cancer progression may closely be associated with the activation of blood clotting system, we hypothesized that D-dimer serves as an indicator for cancer progression. Therefore, we evaluated the role of D-dimer along with CEA as a prognostic and a predictive biomarker in patients with metastatic gastric cancer who had received CTx.

## PATIENTS AND METHODS

### Patients

We retrospectively reviewed all patients newly diagnosed with histologically confirmed metastatic gastric cancer between January 2002 and December 2013 in Gyeongsang National University Hospital (GNUH), Jinju, Korea. Among them, 55 patients treated with palliative first-line CTx and in whom the measurement of pretreatment D-dimer was available were assessed for eligibility. Then, the following patients were excluded: 3 patients with acute illness such as infection within the previous 2 weeks from the time of D-dimer measurement; 2 patients with VTE; 3 patients who were taking anticoagulant medication at the start of CTx; and 1 patient with another malignancy. Finally, a total of 46 patients were included in the study. Institutional Review Board permission of GNUH was obtained for the use of these samples for this analysis (GNUHIRB-2009-19).

### Data Collection

We collected clinical data including age, sex, histological differentiation, site of distant metastasis, response to first-line CTx, survival duration, and the incidence and the type of thromboembolism during CTx. The levels of D-dimer were measured by an immunoturbidimetric method (STA-R Evolution; Diagnostica Stago, Paris, France) and assessed before CTx and at the time of the first response evaluation. Response to first-line CTx was defined according to the Response Evaluation Criteria in Solid Tumor, version 1.1.

### Statistical Analysis

SPSS for Windows, version 21.0 (SPSS Inc., Chicago, IL) was used for all statistical analyses. Categorical variables were presented as frequencies and percentages and compared using the χ^2^ test or Fisher exact test. Continuous variables were presented as mean ± standard deviation and range and compared with Mann–Whitney *U* test and Wilcoxon signed-rank test. Simple regression analysis was performed to convince that the measurements of D-dimer were consistent regardless of the time of D-dimer measurement. No correlation between the D-dimer level and the time of D-dimer measurement was observed (at pretreatment, *r* = +0.048, *P* = 0.750; at the first response evaluation, *r* = −0.148, *P* = 0.357).

The median follow-up duration was calculated by the reverse Kaplan–Meier method. Overall survival (OS) was calculated as the time from the date of the first-line CTx to the date of death from any cause. OS was estimated using the Kaplan–Meier method and comparisons between groups were made using the log-rank test. Multivariate analysis for variables associated with OS via the Cox proportional hazards model was performed and expressed as hazard ratios and 95% confidence intervals. To detect multicollinearity, the variance inflation factor (VIF) was calculated for the variables included in the Cox proportional hazards model. A 2-tailed *P* value of <0.05 was considered to be statistically significant.

According to the minimal *P*-value approach, the cutoff levels of D-dimer and CEA were determined as the value maximizing the log-rank statistic for OS.^18^ Potential cutoff levels between 25 and 75 percentiles in steps of 0.5 μg/mL of D-dimer and in steps of 1.0 ng/mL of CEA were assessed. Finally, 1.5 μg/mL (log-rank statistic 5.51, 1 degree of freedom [df]) and 1.0 μg/mL (log-rank statistic 6.83, 1 df) were decided as the cutoff level of pretreatment D-dimer and as those of D-dimer at the first response evaluation, respectively. *P* values were adjusted by Bonferroni correction for multiple testing. The appropriate cutoff level of CEA, however, could not be determined through the minimal *P*-value approach, because *P* value of <0.05 was not achieved at any level of CEA. Thus, here we used 3.4 ng/mL as the cutoff level of CEA because this value is upper normal limit in our institution. Patients were grouped into low and high D-dimer (or CEA) groups based on these cutoff values.

## RESULTS

### Patients Characteristics

**FIGURE 2:**
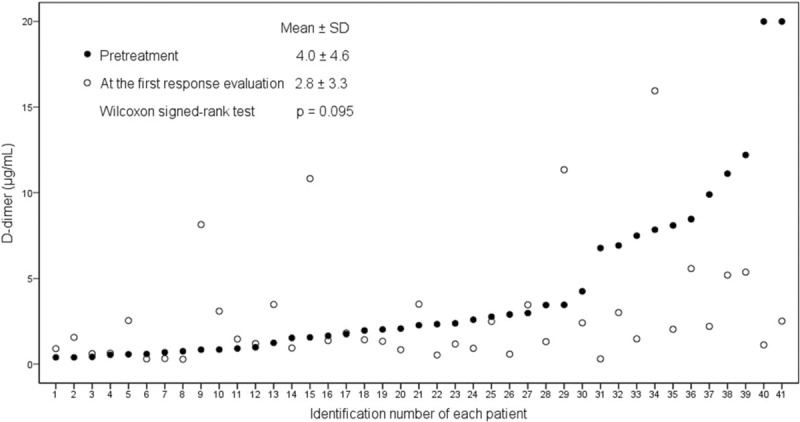
Dot plot with the raw data for the relationship between D-dimer levels at pretreatment and at the first response evaluation.

Baseline characteristics of 46 patients according to pretreatment D-dimer level are shown in Table 1. Median age and distribution of sex were not statistically different between the 2 groups, with male predominance (male and female ratio, 3.6:1). The site of metastasis was similarly distributed between the 2 groups. However, poorly differentiated histology was associated with high pretreatment D-dimer level (*P* = 0.048). The number of CTx cycles was similar between the 2 groups. However, pretreatment D-dimer levels appeared to be higher in the patients showing progressive disease (PD) after CTx, but statistically insignificant. The thrombosis during CTx occurred with similar frequency between the 2 groups. Among 7 patients with thrombosis during CTx, 2 had pulmonary thromboembolism, 1 had deep vein thrombosis of right common femoral vein, 3 had other VTEs (1 left renal vein and 2 portal vein), and 1 had arterial thrombosis of lower abdominal aorta. Figure 2 shows the dot plot with the raw data of each patient for the relationship between D-dimer levels at pretreatment and at the first response evaluation. There was no close relationship between the 2 time points without statistical significance (*P* = 0.095).

**TABLE 1:**
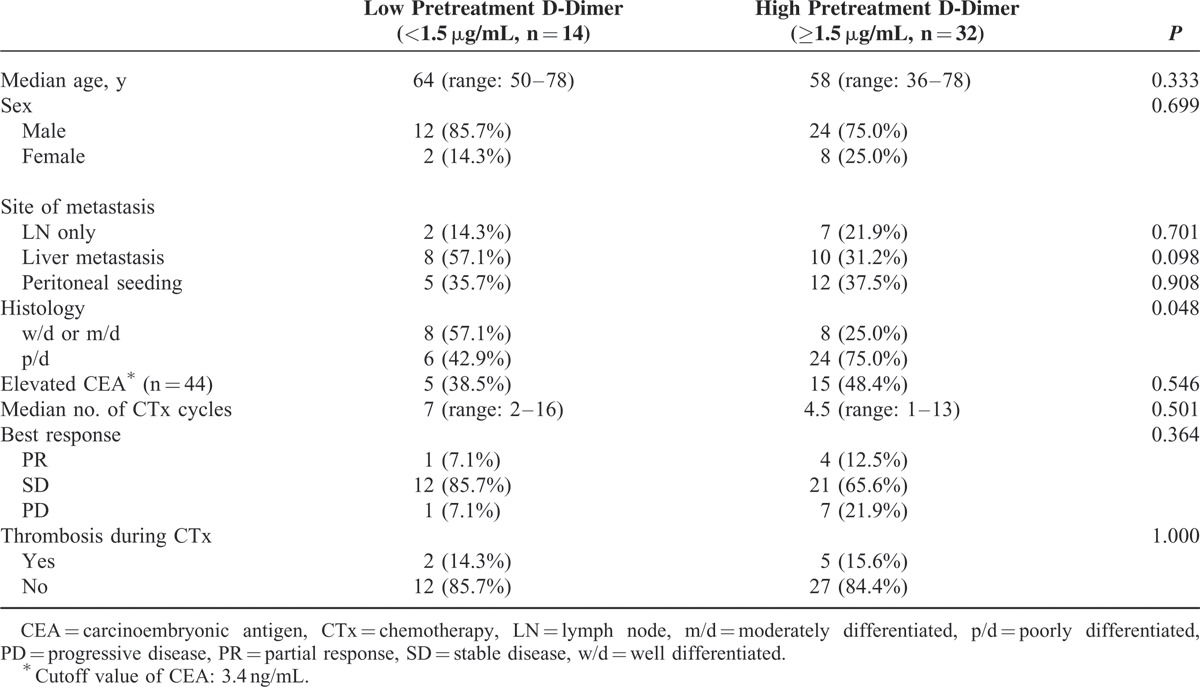
Baseline Characteristics

### Relationship Between the Changes in D-Dimer Levels and the Responses to CTx

The changes in D-dimer levels between the pretreatment and the first treatment response evaluation were assessed in 41 patients available for response evaluation (Table 2). At the first response evaluation, the mean level of D-dimer was significantly decreased by 2.11 μg/mL in 34 patients either with partial response (PR) or stable disease (SD) (*P* = 0.011). The mean D-dimer levels of 8 patients with PR and 26 patients with SD were decreased by 3.11 and 1.80 μg/mL compared to that of pretreatment D-dimer, respectively (*P* = 0.093 and *P* = 0.055). In contrast to PR or SD, in 7 patients with PD, the mean level of D-dimer at the first response evaluation was increased by 2.46 μg/mL although the difference did not reach statistical significance (*P* = 0.176). These results suggest that D-dimer level may serve as a predictive biomarker to CTx.

**TABLE 2:**
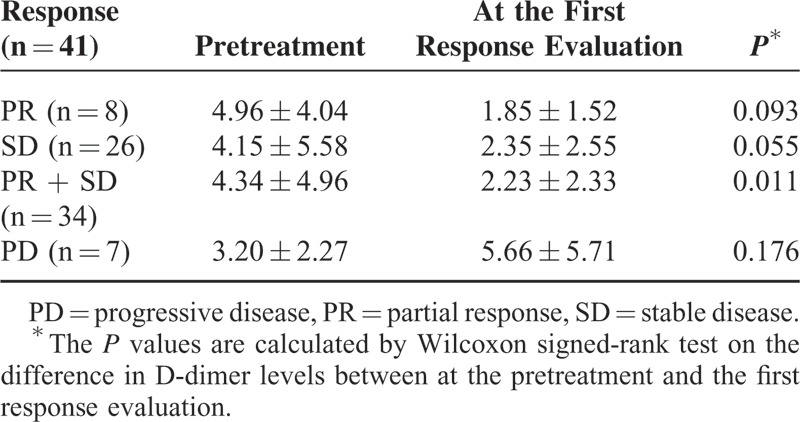
Difference of D-Dimer Levels

### Survival Analysis

**FIGURE 3:**
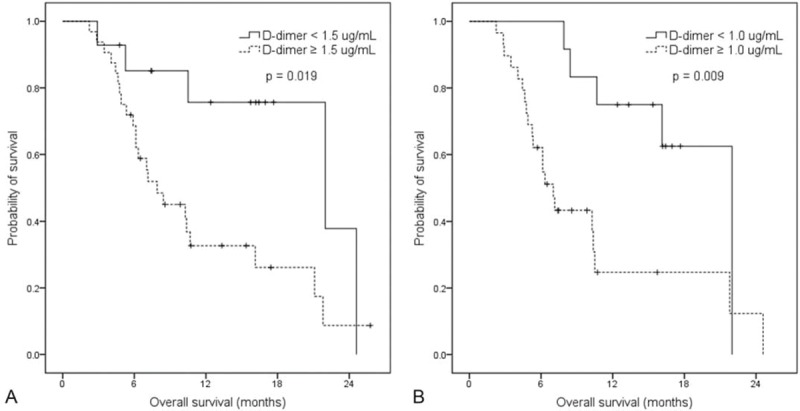
OS curve by D-dimer levels. (A) Patients with D-dimer levels <1.5 μg/mL at the pretreatment showed a significantly longer OS than those with D-dimer levels ≥1.5 μg/mL (median OS, 22.0 vs 7.9 mo, respectively). (B) Patients with D-dimer levels <1.0 μg/mL at the first response evaluation showed a significantly longer OS than those with D-dimer levels ≥1.0 μg/mL (median OS, 22.0 vs 7.0 mo, respectively). OS = overall survival.

Median follow-up duration was 16.2 months (range: 2.2–25.8 months) and median OS was 10.5 months. In survival analysis, the group with high pretreatment D-dimer level was associated with worse survival than that with low pretreatment D-dimer level (median OS, 22.0 vs 7.9 months, *P* = 0.019, *P* = 0.171 after Bonferroni correction; Figure 3A). The patients with high D-dimer level (≥1.0 μg/mL) at the first response evaluation also showed shorter OS than those with low D-dimer (<1.0 μg/mL) (median OS, 22.0 vs 7.0 months, *P* = 0.009, *P* = 0.045 after Bonferroni correction; Figure 3B).

**FIGURE 4:**
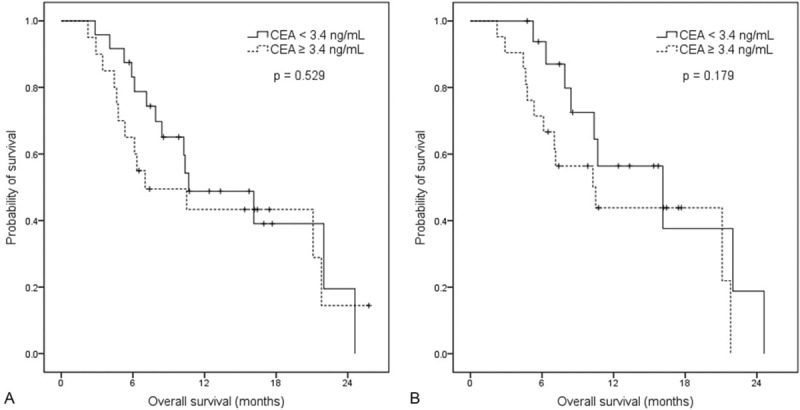
OS curve by CEA levels. There was no significant difference in OS between low and high CEA groups (A) at the pretreatment (median OS, 10.7 vs 7.0 mo, respectively) and (B) at the first response evaluation (median OS, 16.1 vs 10.5 mo, respectively). CEA = carcinoembryonic antigen, OS = overall survival.

For the survival analysis according to CEA levels at pretreatment and the first response evaluation, survival curves was not different between low and high CEA level at pretreatment evaluation (median OS, 10.7 vs 7.0 months, *P* = 0.529; Figure 4A) and at the first response evaluation (median OS, 16.1 vs 10.5 months, *P* = 0.179; Figure 4B). This result suggests that pretreatment CEA level could not serve as a prognostic biomarker in metastatic gastric cancer.

Univariate analysis showed that high level of D-dimer both at the pretreatment and at the first response evaluation were unfavorable prognostic factors along with poor response to CTx and poorly differentiated histology. Multivariate analysis for these factors demonstrated that high level of D-dimer at the first response evaluation and poorly differentiated histology were independent unfavorable prognostic factors (Table 3). The estimated VIFs were <2 for all variables included in the multivariate analysis, indicating that multicollinearity was not a problem. These results suggest that high level of D-dimer at the first response evaluation may have more significant influence on survival than those at pretreatment evaluation.

**TABLE 3:**
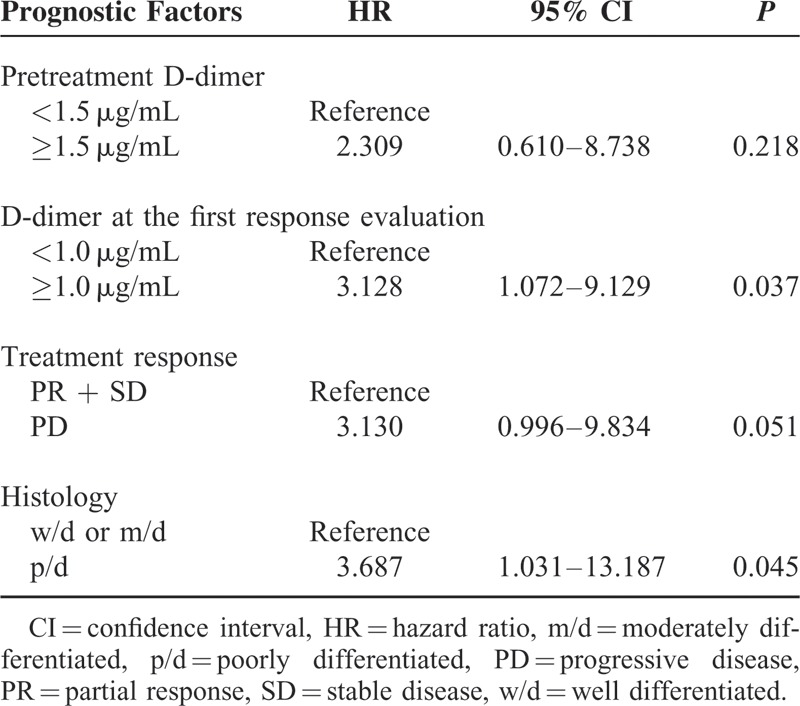
Multivariate Analysis for Overall Survival

## DISCUSSION

We evaluated the value of D-dimer as a prognostic and predictive biomarker for CTx along with CEA in metastatic gastric cancer. This study showed that patients with high level of D-dimer at pretreatment had poor prognosis, but not those with high level of CEA. There were several reports on the relationship between D-dimer level and prognosis in patients with gastric cancer, but these studies were performed in patients with operable gastric cancer.^19,20^ This study has unique value that the role of D-dimer was shown as a prognostic factor in metastatic gastric cancer. Furthermore, this study revealed that high level of D-dimer at the first response evaluation was more significantly associated with poor prognosis than those at pretreatment. These findings suggest that the prognosis can change according to whether the level of D-dimer decreases <1.0 μg/mL during anticancer therapy in patients with metastatic gastric cancer.

The clinical importance of the change of D-dimer level during anticancer therapy was further supported by the analysis for the association between the therapeutic response and the change of D-dimer level shown in this study. There has been no report that evaluated the role of D-dimer as a predictive biomarker for response to anticancer therapy in gastric cancer. The present study revealed that D-dimer level in patients with PR and SD tended to decrease at the first response evaluation with a greater degree in those with PR, and that D-dimer level in patients with PD showed an increasing tendency at the first response evaluation. Although the degree of changes in D-dimer levels in patients with PR was greater than those in patients with either PR or SD, the change of D-dimer level was not statistically significant in patients with PR. This may result from the decreased statistical power derived from the decrease in the number of patients. The role of D-dimer as a predictor for therapeutic response has been recently reported on other malignancies. Inanc et al^21^ reported that when comparing D-dimer level at pretreatment and after 3 cycles of CTx in patients with colorectal cancer, D-dimer level in those with PR was significantly decreased, whereas in case of PD, considerable increase was seen in D-dimer level. Komurcuoglu et al^22^ also demonstrated that pretreatment D-dimer level in patients with lung cancer was significantly higher in nonresponders than in responders. These reports showing a tendency to be consistent with the result of present study support the role of D-dimer as a predictor for therapeutic response in cancer patients. This study is the first report that suggests the clinical significance of D-dimer as a predictor for therapeutic response in patients with metastatic gastric cancer.

In the present study, we found that poorly differentiated histology was associated with poor survival and high level of pretreatment D-dimer. The correlation between histological differentiation and D-dimer has not been reported in metastatic gastric cancer, but there are some related reports. Diao et al^23^ showed that the D-dimer level was higher in histological grades 3 and 4 than in histological grades 1 and 2 in patients with esophageal cancer. Furthermore, Hwang et al^24^ reported that 80% of advanced gastric cancer patients with disseminated intravascular coagulation had signet-ring cell and poorly differentiated histology. In contrast, there is a report^25^ showing no correlation between these 2 factors in colon cancer. Therefore, further studies will be needed for the relationship between D-dimer and histological grade.

Regarding the determination of cutoff value in this study, it is debatable to apply the different cutoff value of D-dimer in the same patients during the treatment course. Ay et al^26^ reported a significant difference in survival rate according to the D-dimer level in a prospective study performed on cancer patients. Particularly, a group of >1.33 μg/mL of D-dimer had the poorest prognosis. Liu et al^19^ evaluated the prognosis according to preoperative level of D-dimer in 247 patients with gastric cancer and showed a significant difference in OS based on cutoff value of 1.465 μg/mL for D-dimer. These cutoff values are between the values of the pretreatment (1.5 μg/mL) and the first response evaluation (1.0 μg/mL) in our study. Additional study is warranted for optimal cutoff value of D-dimer in cancer patients.

In contrast to D-dimer, CEA has neither prognostic nor predictive biomarker role in metastatic gastric cancer in the present study. Lu et al^27^ could not find the role of CEA as a prognostic factor in metastatic gastric cancer, either. In addition, Blackwell et al^15^ reported that changes in D-dimer levels were correlated more strongly with disease progression than changes in CEA levels in colorectal cancer. At present, the impact of CEA on prognosis in patients with gastric cancer has not been established.

There are several limitations in this study. First, this study is retrospective in nature. Second, subject enrolment period was long and small number of patients was included in this study. In part, it was because of the fact that the measurement of D-dimer had not been considered as a part of baseline assessments in patients with metastatic gastric cancer.

In conclusion, this study suggests that high D-dimer levels at pretreatment and the first response evaluation are associated with poor prognosis in patients with metastatic gastric cancer, and that D-dimer also serves as a predictive biomarker for therapeutic response. These findings provide evidence to support that the activation of blood coagulation system is closely related to cancer progression and prognosis. Large-scale prospective study is warranted to validate the role of D-dimer in patients with metastatic gastric cancer.
